# Assessing Gastrointestinal Tolerance in Healthy Adults: Reliability and Validity of a Weekly Questionnaire

**DOI:** 10.1016/j.cdnut.2023.101976

**Published:** 2023-07-20

**Authors:** Melissa Alvarez, James Colee, Bobbi Langkamp-Henken, Wendy J. Dahl

**Affiliations:** 1 Food Science and Human Nutrition Department, University of Florida, Gainesville, FL, United States; 2 Institute of Food and Agricultural Sciences (IFAS) Statistics, University of Florida, Gainesville, FL, United States

**Keywords:** gastrointestinal symptoms, nondigestible carbohydrates, gastrointestinal tolerance, stool form, stool frequency

## Abstract

When examining gastrointestinal tolerance to nondigestible carbohydrates, a weekly vs. daily symptoms questionnaire may lessen participant burden. This secondary analysis examined the reliability and validity of the Gastrointestinal Symptom Rating Scale (GSRS) in healthy adults. The internal consistency reliability of the GSRS syndromes and a daily questionnaire (DQ) comparator were determined. The GSRS syndromes prediction of slow transit stool form was assessed by ROC analysis. The DQ (*α* = 0.76) and GSRS syndromes of constipation (*α* = 0.73; *ω* = 0.74), and diarrhea (*α* = 0.76; *ω* = 0.77) exhibited acceptable reliability, as did the GSRS overall (*α* = 0.76; *ω* = 0.87) but not the syndromes of abdominal pain (*α* = 0.54; *ω* = 0.54), reflux (*α* = 0.69; *ω* = 0.67), or indigestion (*α* = 0.64; *ω* = 0.67). The GSRS syndromes predicted slow transit stools (AUC = 0.855), and the GSRS items of stomach pain, nausea, flatus, constipation, and diarrhea were moderately correlated (*ρ* = 0.55–0.64; *P* < 0.001) with the corresponding DQ items. The GSRS may be useful to assess gastrointestinal tolerance and efficacy of nondigestible carbohydrates given its performance at predicting slow transit stools, suggestive of constipation.

## Introduction

Current fiber intakes fall far short of recommendations [1], and consuming foods with added fiber has been suggested to increase total fiber intakes to recommended levels [2]. Isolated and synthesized nondigestible carbohydrates require demonstrated evidence of health benefits in healthy individuals before inclusion as fiber on food labels in the United States [3]. Beyond efficacy testing, consumer acceptance is essential to ensure that food products with added fiber will be consumed. In contrast, gastrointestinal intolerance to these foods may impede increased fiber intake. Given that gut microorganisms variably ferment nondigestible carbohydrates, these food ingredients differ in the intensity of gastrointestinal symptoms induced [4] and thus require assessment of tolerance. In studies assessing gastrointestinal tolerance to nondigestible carbohydrate ingredients, daily reporting of symptoms is often undertaken [[5], [6], [7]], which poses a time burden for participants. Other studies have relied on weekly [8] or less-frequent assessments at baseline and the end of the intervention period [9]. However, the validity and reliability of such tools are often not described.

Although developed for patient populations, the Gastrointestinal Symptom Rating Scale (GSRS) may be useful for gastrointestinal tolerance testing. The GSRS was first developed for use in patients with peptic ulcer disease [10] and later demonstrated adequate reliability and validity in patients with gastroesophageal reflux disease [11] and renal transplant patients [12,13]. The GSRS is a weekly questionnaire that assesses the severity of gastrointestinal symptoms an individual has experienced over the past week [14]. The questionnaire consists of 15 questions contributing to 5 syndromes, including abdominal pain (stomach pain, hunger pains, and nausea), reflux (heartburn and acid reflux), indigestion (rumbling, bloating, burping, and flatulence), constipation (constipation, hard stools, and the feeling of incomplete evacuation), and diarrhea (diarrhea, loose stools, and urgent need for defecation). It is scored from 1 (no discomfort at all) to 7 (very severe discomfort).

The GSRS has been utilized in healthy adults to assess differences in gastrointestinal symptoms with added fiber intake [15] and probiotic supplementation [16], as well as comparing healthy controls with adults with slow and normal transit constipation [17]. Additionally, it has been used to assess gastrointestinal symptoms reported by adults with depression [18] and after gastric bypass surgery [19]. Given the potential to use a weekly questionnaire to assess gastrointestinal symptoms in populations free of gastrointestinal disease, additional validity testing is needed. This study aimed to determine the validity and reliability of the GSRS in a healthy adult population. As interventional trials examining the efficacy of nondigestible carbohydrates commonly assess gastrointestinal function, a secondary aim was to test the performance of GSRS for predicting slow transit stool form.

## Methods

This secondary analysis used de-identified baseline (preinterventional) data from 6 interventional studies [16,[20], [21], [22], [23], [24]] to determine whether the GSRS demonstrated validity and reliability in a healthy adult population. The study was approved as exempt by the University of Florida’s Institutional Review Board (IRB201701465). Interventional studies with a minimum of one baseline GSRS were included. For assessing daily gastrointestinal symptoms (bloating, flatulence, abdominal cramping, stomach noises, nausea), 7 days of reporting at baseline using a Likert scale (0 = none, 3 = mild, 6 = very severe), as typically used in such studies [25], was required. Daily reporting of Bristol Stool Form (BSF) [26] and bowel movement frequency, if available, were also included.

The means of GSRS syndrome scores were calculated from their respective items. For the 5 daily gastrointestinal symptom questions (DQ), a 7-day mean was calculated. Convergent validity was established for the daily questionnaire and the GSRS symptom items and syndromes using Spearman correlation (*ρ*) and internal consistency reliability by Cronbach’s alpha and McDonald's omega. To determine an optimal prediction model using all GSRS syndromes (as an assessment of concurrent validity), a boosted regression tree was estimated to predict slow transit stools (any reporting of BSF 1 or 2). Area under the curve (AUC) from ROC analysis was used as a measure of GSRS syndrome performance and was interpreted as <0.7 poor, 0.7–0.8 acceptable/fair, 0.8–0.9 excellent, and >0.9 outstanding. Sensitivity was defined as the ability of the GSRS syndrome scores to correctly identify those reporting slow transit stools by BSF types 1 or 2. Specificity was the ability of the GSRS syndrome scores to correctly identify those not reporting BSF types 1 or 2. Spearman’s correlation was conducted to assess correlations between individual GSRS symptom ratings (stomach pain, nausea, flatus, constipation, and diarrhea) and the continuous variables of DQ ratings of similar constructs, mean BSF, and bowel movement frequency. Statistical analysis was accomplished with JMP v17. Significance was set at *P* = 0.05.

## Results

**FIGURE 1:**
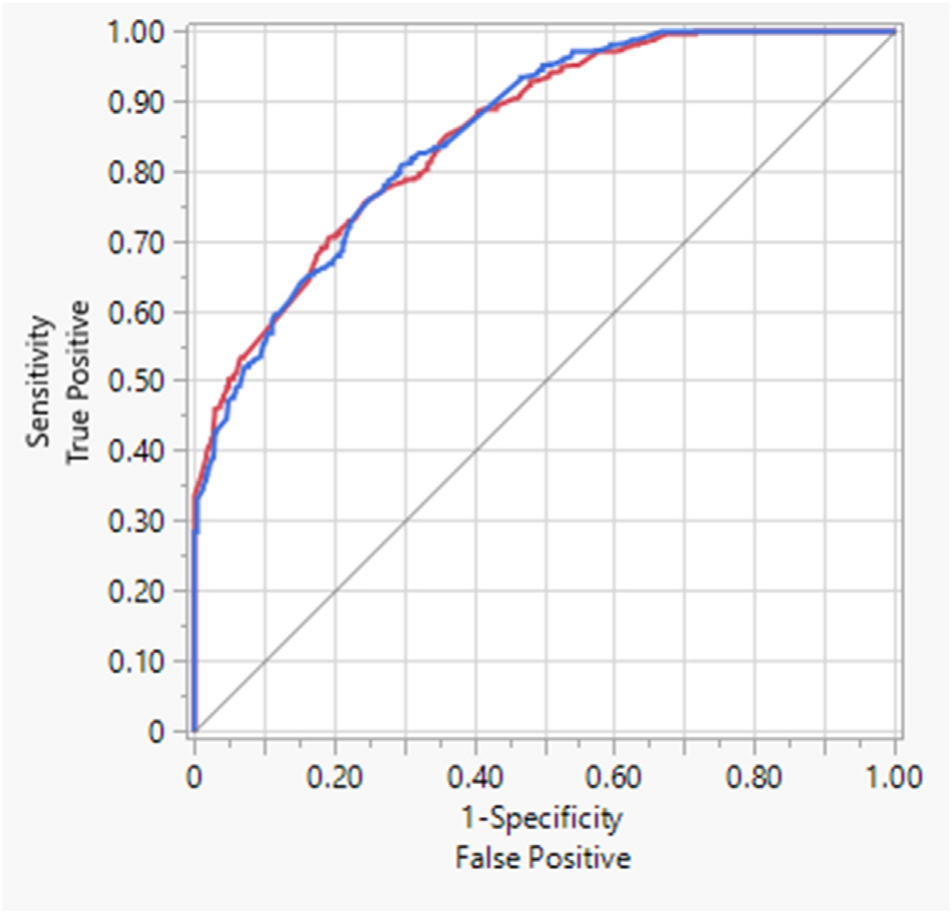
Receiver-operating characteristic (ROC) curve of the optimal prediction model of the Gastrointestinal Symptom Rating Scale syndromes using a boosted regression tree estimation to predict slow transit stools (any reporting of BSF 1 or 2) in healthy adults. Area under the curve (AUC) = 0.855. Blue and red lines indicate “slow transit” and “no slow transit,” respectively, with equal prediction.

GSRS data were available for 1050 subjects, with subsets of data for stool frequency (*n* = 964), BSF (*n* = 886), and DQ (*n* = 80). The mean age of participants was 25.3 ± 11.4 y (range: 18–65 y), with a BMI of 24.6 ± 5.1 kg/m^2^. For Cronbach’s alpha, the GSRS syndromes of abdominal pain (*α* = 0.54), reflux (*α* = 0.69), and indigestion (*α* = 0.64) fell below the threshold of acceptable reliability, whereas constipation (*α* = 0.73), and diarrhea (*α* = 0.76), and the overall tool (*α* = 0.76) exhibited acceptable reliability as did the 5 items of the DQ (*α* = 0.76). McDonald's omega for the overall GSRS was 0.87; whereas the syndromes of abdominal pain (*ω* = 0.54), reflux (*ω* = 0.67), and indigestion (*ω* = 0.68) were below the threshold, and constipation (*ω* = 0.74) and diarrhea (*ω* = 0.77) demonstrated acceptable internal consistency. The optimal prediction model using all GSRS syndromes used to predict slow transit stools (any reporting of BSF 1 or 2) resulted in an AUC of 0.855 (Figure 1), with reflux syndrome contributing the most to its performance. Alone, GSRS constipation syndrome predicted BSF 1 or 2 with an AUC of 0.551. Table 1 shows the correlations between select items of the GSRS and the DQ items assessing similar constructs. Additionally, mean BSF was moderately but significantly (*ρ* = 0.55; *P* = 0.001) correlated with bowel movement frequency.

**TABLE 1:** Association between select GSRS questions and daily gastrointestinal symptom reporting

GSRS questions	Mean weekly DQ items	Spearman’s correlation (*ρ*)	*P* value
Q1 Stomach pain	DQ Abdominal cramping	0.59	<0.001
Q5 Nausea	DQ Nausea	0.62	<0.001
Q9 Flatus	DQ Flatulence	0.64	<0.001
Q10 Constipation	DQ Constipation	0.61	<0.001
Q11 Diarrhea	DQ Diarrhea	0.55	<0.001

Abbreviations: DQ, daily gastrointestinal symptom questionnaire; GSRS, Gastrointestinal Symptom Rating Scale; Q, question.

## Discussion

This analysis aimed to elucidate the elements of validity and reliability of the GSRS in a healthy adult population and thus inform its use as a weekly questionnaire for gastrointestinal symptom tolerance testing in dietary interventions. The overall GSRS items and constipation and diarrhea syndromes exhibited adequate internal consistency reliability in the healthy adult population data, but the abdominal pain syndrome of the GSRS is not a reliable syndrome score in a healthy population. This is not surprising given that nausea is grouped with stomach and hunger pain, seemingly unrelated symptoms in healthy individuals. Although the constipation syndrome was poor, a prediction model using all GSRS syndromes was an excellent predictor of slow transit stool form. This finding is particularly applicable, as stool form is often an outcome of clinical trials testing the efficacy of nondigestible carbohydrates to enhance laxation. Individual questions of the GSRS were moderately correlated with daily querying of gastrointestinal symptoms. Thus, daily reporting, which may add to participant burden, may not be required if the weekly GSRS is utilized. Components of the GSRS may be appropriate for studying the effect of nondigestible fibers, dietary patterns, and dietary supplement interventions on gastrointestinal wellness in healthy populations.

The mean daily bowel movement frequency was moderately correlated with the mean BSF. This finding agrees with the self-reporting of “usual or most common” BSF type and stool frequency in a nationally representative sample [27]. Although often reported in the literature [6,23], mean BSF may not be the most appropriate interpretation of stool form as individuals can experience slow transit and fast transit stools during the period of assessment, even for the same bowel movement episode [28]. Such individuals will be miscategorized, that is, as having normal average stool form, when instead, they may be alternating between BSF-type extremes. Other researchers have used dichotomous categories such as BSF < 3, representing hard stools, and BSF > 3, a regular bowel habit [29], or simply BSF of 3–5 as normal bowel function [30]. Alternatively, tri-nominal categories of BSF slow (types 1 and 2), normal (types 3–5), and fast transit (types 6 and 7) have been used for stool form reporting [15,28]. This interpretation suggests 1 category as “satisfactory” or “healthful” (a proxy of normal transit) and 2 categories as “unsatisfactory” or “unhealthful” (a proxy of slow and fast transit), which may be most appropriate for assessing wellness. Alternatively, a revised scale centered at zero [new score = absolute value (BSF score minus 4)] and averaging the new value may be suitable for a sample or population measure of stool form.

This assessment of the reliability and validity of the GSRS had several limitations. Data were from self-report and, thus, may lack reliability. Specifically, for BSF, expert ratings by multiple external observers may strengthen reliability. Additionally, symptom reporting by healthy individuals may differ from patients with a diagnosis of gastrointestinal disease or condition, as their attitudes toward symptoms may differ, as may the impact on quality of life. Thus, when evaluating tolerance of dietary interventions in healthy subjects, an assessment of quality of life, such as the Digestion-associated QOL Questionnaire (DQLQ), designed to assess physical and mental wellbeing changes due to gastrointestinal symptoms [31], may be important to include in addition to tracking frequency and intensity of symptoms. Furthermore, structural validity by factor analysis and test–retest reliability of the GSRS were not examined. However, Spearman’s correlation analysis evaluated the convergent validity, and the prediction tree performance analysis tested the discriminative validity. The construct validity of the GSRS has been established in patient populations [11,12], and application to the healthy population may be appropriate. Only 5 items of the DQ against GSRS items were tested. Additional concurrent validation could be undertaken to test the remaining GSRS items with daily ratings as comparators and the construct validity of items used to rate daily gastrointestinal symptoms. Although expected in healthy subjects, the low incidence of high stool frequency diarrheal symptoms and BFS types 6 and 7 precluded the testing of the performance of GSRS diarrhea syndrome. Finally, given the availability of digital diaries and applications using smartphones, symptom scoring can be administered multiple times per day [32], and thus, the utility of daily or weekly questionnaires may be questioned. However, research is needed to compare these methodologies, specifically assessing the potential participant burden of immediate symptom reporting compared with daily or weekly surveys in longer-term trials, and whether this burden negatively impacts the reliability and validity of such measures. Although recall bias may be minimized with immediate symptom reporting, response fatigue and potentially, intrusiveness bias may impact gastrointestinal symptom reporting [33].

## Conclusion

This study aimed to determine the reliability and validity of the GSRS in healthy adults and its ability to predict slow transit stool form indicative of constipation. The GSRS syndromes of constipation and diarrhea showed acceptable internal consistency reliability, and the tool demonstrated concurrent validity when compared with daily reporting of symptoms. Furthermore, the symptoms and syndromes exhibited adequate reliability. The GSRS predicted slow transit stools, a common sign of constipation, and thus may be a quick and useful weekly tool to assess gastrointestinal tolerance of nondigestible carbohydrates.

## Funding

This study was supported by the Department of Food Science and Human Nutrition, University of Florida/Institute of Food and Agricultural Sciences.

## Data availability

Data analyzed in this manuscript will be made available upon request and pending approval.

## Author contributions

The authors’ responsibilities were as follows – WJD and BL performed the original studies; MA, JC, WJD contributed to the conception and design of the study; JC analyzed the data; MA, WJD wrote the original draft; MA, JC, BL, WJD reviewed and edited the manuscript; and all authors: read and approved the final manuscript.

## Conflict of interest

The authors report no conflicts of interest.
